# Do Different Models Induce Changes in Mortality Indicators? That Is a Key Question for Extending the Lee-Carter Model

**DOI:** 10.3390/ijerph18042204

**Published:** 2021-02-23

**Authors:** Ana Debón, Steven Haberman, Francisco Montes, Edoardo Otranto

**Affiliations:** 1 Centro de Gestión de la Calidad y del Cambio, Universitat Politècnica de València, Camino de Vera s/n, E-46022 Valencia, Spain; 2 Cass Business School, University of London, London EC1Y 8TZ, UK; S.Haberman@city.ac.uk; 3 Department of Statistics and Operations Research, Universitat de València, E-46100 Burjassot, Spain; montes@uv.es; 4 Department of Economics, Università di Messina, 98122 Messina, Italy; edoardo.otranto@unime.it

**Keywords:** Lee-Carter models, block-bootstrap, functional ANOVA, forecasting, mortality indicators

## Abstract

The parametric model introduced by Lee and Carter in 1992 for modeling mortality rates in the USA was a seminal development in forecasting life expectancies and has been widely used since then. Different extensions of this model, using different hypotheses about the data, constraints on the parameters, and appropriate methods have led to improvements in the model’s fit to historical data and the model’s forecasting of the future. This paper’s main objective is to evaluate if differences between models are reflected in different mortality indicators’ forecasts. To this end, nine sets of indicator predictions were generated by crossing three models and three block-bootstrap samples with each of size fifty. Later the predicted mortality indicators were compared using functional ANOVA. Models and block bootstrap procedures are applied to Spanish mortality data. Results show model, block-bootstrap, and interaction effects for all mortality indicators. Although it was not our main objective, it is essential to point out that the sample effect should not be present since they must be realizations of the same population, and therefore the procedure should lead to samples that do not influence the results. Regarding significant model effect, it follows that, although the addition of terms improves the adjustment of probabilities and translates into an effect on mortality indicators, the model’s predictions must be checked in terms of their probabilities and the mortality indicators of interest.

## 1. Introduction

In the context of rapid recent demographic changes, such as the finding that “human senescence has been delayed by a decade” in [1], the development of new models for fitting and projecting life tables is a key major direction of research for demographers, actuaries, epidemiologists and other biomedical researchers. Even if new proposals such as [2] are emerging, different extensions of the seminal Lee-Carter model introduced by [3] are still being developed. Thus recently, [4] compared three probability models (Poisson, Negative Binomial and Binomial) based on the Generalized Linear Model (GLM) framework of the Lee-Carter model. In fact, the majority of the existing models proposed in the actuarial and demographic literature fall into an age/period/cohort framework that builds on the Lee-Carter model [5].

It is also essential to have an appropriate set of indicators for studying these phenomena, including life expectancy, the Gini index, and the modal age at death [6,7]. All of these indicators can be projected using the predictions of annual age-specific mortality probabilities, $q_{xt}$, obtained from different methodologies, which are based, in this paper, on Lee-Carter models. The errors associated with these estimations can be calculated employing a block-bootstrap methodology [8], and confidence intervals can be provided.

This paper aims to evaluate whether improvement in models will be reflected in significant differences between the projections of these mortality indicators. Therefore, we consider the Lee-Carter model with one or two temporal terms, and we also consider the addition of a cohort effect. Authors such as [9,10] find there is little change in the ability to forecast life expectancy in comparison with the original Lee-Carter model. There has been extensive work on the number of components to be included in Lee-Carter type models. [11] conclude that there is no real penalty for adding extra terms and recommend using six components, using the results of [12] for providing some theoretical reasons for this conclusion. [13,14,15] are extensive studies which compared many models and countries. Consequently, using only two-period components in Lee-Carter type models could seem inadequate in the light of these empirical and theoretical results, but we aim to investigate the statistical methodology for comparing the forecasts of mortality indicators. The methodology can be extended to any forecasting results.

Our study provides two important innovations which are significant contributions to the existing literature on mortality modelling. First, we use the block-bootstrap technique of [8] to produce the confidence intervals for the mortality indicators allowing for parameter error. We extend this by testing whether the forecasts are affected using the block-bootstrap from fitting mortality models other than those used to forecast the indicator. Our second methodological innovation is the use of functional analysis techniques to detect differences between the projections of the mortality indicators. This is important because the projections of an indicator over time will consist of correlated values, which requires either the use of longitudinal data techniques or functional data methods to analyze precisely.

The paper is structured as follows. Material and methods are described in Section 2. Then, Section 2.1 gives a brief summary of Lee-Carter models and Section 2.2 discusses the indicators of mortality used in this analysis. The following Section 2.3 introduces the block-bootstrap techniques for estimating prediction intervals. Functional data analysis techniques allowing the comparison of mortality indicators are then discussed in Section 2.4. Later, in Section 3 we present results of our analysis of Spanish mortality data by means of the methods and indicators discussed previously. Finally, Section 4 draws conclusions from these results and summarises our findings.

## 2. Materials and Methods

Mortality is one of the demographic components that began to be studied as early as the 17th century in England, but today it presents challenges that are more relevant than ever. The main tool to study mortality is the life tables. Mortality rates are often presented in the form of life tables, giving the probabilities of dying at each age (conditional on survival to that age) for a population. There are two types of life tables: cohort and period tables. The cohort life table presents the mortality experience of a specific cohort (born in the same year), and hence it reflects the actual mortality rates experience from birth until all individuals in the cohort have died. In contrast, the period life table presents what would happen to a synthetic cohort if it experienced the mortality conditions of a particular time period throughout its entire life. Cohort tables therefore require data over many years and will only be complete on exhaustion of the cohort. Because of this, we normally use mortality indicators based on the period life table.

**Figure 1:**
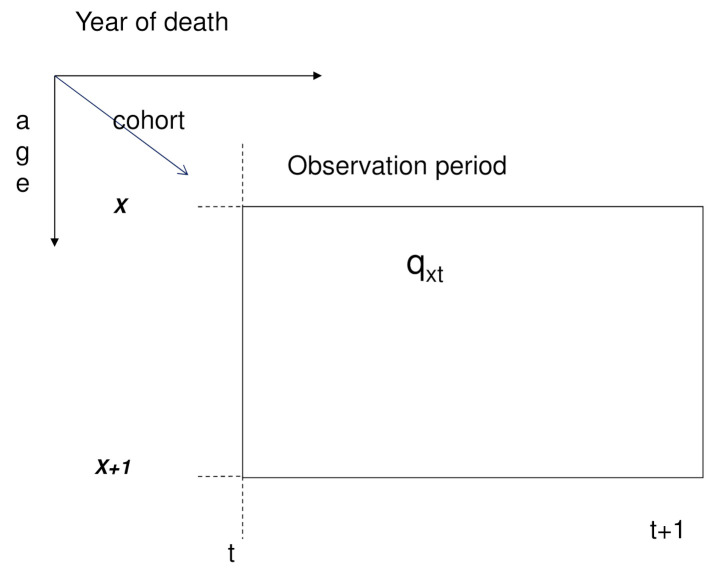
Illustration of age, cohort and period dimensions.

Crude mortality probabilities at age *x* and time *t* are typically based on the corresponding number of deaths recorded, $d_{xt}$, relative to the population aged *x* last birthday at the start of the calendar year, i.e., the initial exposure-to-risk, $E_{xt}$. Typically, it is assumed that these crude probabilities are random realisations of a smooth underlying function of age, period and birth cohort, which can be found by statistical analysis. Static life tables assume that the mortality probabilities do not change with time and so are functions of age only. In contrast, dynamic life tables are rectangular arrays of mortality probabilities, $\left(q_{xt}\right)$, with each column in this array representing the period life table for year *t* (Figure 1). See [16] for a more detailed discussion of dynamic life tables.

### 2.1. Lee-Carter Models

The Lee-Carter model was introduced in [3] and has become the standard model for projecting dynamic life tables. The model was used with the logarithm of the annual age-specific central mortality rates, $m_{xt}$, which are equivalent to the force of mortality, $\mu_{xt}$, under the assumption that $\mu_{xt}$ is constant over integer ages and years. For the calculation of mortality indicators, the projected central mortality rates are converted into annual age-specific probabilities of death using standard formulae. In contrast, this paper models the annual age-specific probability of death, $q_{xt}$. Therefore, this approach has the advantage that the indicators studied in this paper can be calculated directly.

In [3], least-squares estimation via singular value decomposition of the matrix of the log age-specific central mortality rates was used to fit the model. This implicitly assumed that the death counts are homoscedastic across ages and years. In contrast, the maximum-likelihood method, assuming that death counts follow the Poisson distribution, avoids this drawback [17]. However, using the Poisson model for the death counts is inconsistent with our desire to model the age-specific probabilities of death, and so instead we model death counts as binomial random variables. In addition, [18] compared Poisson models for the force of mortality and binomial models for probabilities of death for six different countries and, in most of them, the binomial models outperform the Poisson ones. However, the results using the binomial model and probabilities of death show evidence of overdispersion, since the deviance statistic is greater than the degrees of freedom in the model. Therefore, in this study, the quasi-binomial family model is used to model the probabilities of death, which overcomes this problem. To do this, we use the extended version [19,20] of the Lee-Carter predictor structure in conjunction with the logit transformation of the probability of death, $q_{xt}$, i.e., we use

(1) $$\ln\left(\frac{q_{xt}}{1-q_{xt}}\right)=a_{x}+\sum_{i=1}^{r}b_{x}^{\left(i\right)}k_{t}^{\left(i\right)}+\epsilon_{xt}.$$

In our application with Spanish mortality data, we have used (Equation (1)) with r=1 (i.e., the classical Lee-Carter structure) and r=2 (as investigated in [20]), and consequently the corresponding models will be denoted *LC1* and *LC2*, respectively. The estimation of the parameters for these models is carried out by means of maximum likelihood methods assuming the quasi-binomial distribution for the death counts, using the gnm library published by [21] in R [22]. A detailed description of these models and the alternative method to fit them to data can be found in [23]. Besides, the fitting procedure of a range of different extensions to the Lee-Carter model using the gnm library from [21] can be found in [18].

In addition to extending the Lee-Carter model with additional age/period terms, various authors have proposed modifying the Lee-Carter model to include the influence of the year of birth (cohort) c=t−x. In [24], a model *H1* is analyzed and recommended as the simplest version of the Lee-Carter model with a cohort term [25]. We use this *H1* model with the logit transformation of the probability of death $q_{xt}$ to give,

$$\ln\left(\frac{q_{xt}}{1-q_{xt}}\right)=a_{x}+b_{x}k_{t}+\gamma_{t-x}+\epsilon_{xt},$$

which will also be referred to as *H1* in this paper.

To forecast $q_{xt}$, we first model $k_{t}^{\left(i\right)},\;i=1,2$ and $\gamma_{t-x}$ as time series using the Box-Jenkins methodology. Separate univariate ARIMA processes are applied to the first two-period components of the *LC2* model, due to the underlying assumption that $k_{t}^{\left(1\right)}$ and $k_{t}^{\left(2\right)}$ are independent. This is a consequence of using an orthogonal decomposition [26].

Finally, we note that many studies (for instance [10,18,27]) have found that although the introduction of a cohort term to the Lee-Carter models generally leads to an improvement in overall fit, models may become less robust when fitting it to data and cause problems when forecasting mortality rates. To overcome this, we have implemented in Section 3.1 a two stage fitting procedure, as suggested in [25]. According to [28] the two-stage approach will generally lead to more imperfect fits to the available data than a one-stage approach and are therefore not proper maximum likelihood estimates. However, the potential loss of goodness of fit of the two-stage approach may be justified if it gives a model that is more robust to changes in the data [29].

### 2.2. Mortality Indicators

When projecting dynamic life tables, it is important to be able to summarise the changes in mortality rates across different ages. In previous studies, such as [10,13,14,24], the key indices of interest have been mortality rates, life expectancies and discounted annuity values. The last two of these are indicators related to the typical life span, and are especially important in an actuarial context. However, for an accurate assessment of risk, measures of the dispersion of life spans are also important. In this paper, we study three period-based mortality indicators: life expectancy and modal age of death (which are both measures of typical life span in a population) and the Gini index (to measure the dispersion of life span). Each of these mortality indicators can be calculated from a dynamic period life table, as described in [6], and they are briefly described here below.

The life expectancy for individuals with age *x* is given by

$$e_{xt}=\frac{T_{xt}}{l_{xt}},$$

where $l_{xt}$ is the hypothetical number of people alive from the synthetic cohort experiencing mortality rates for year *t* at the beginning of the age interval [x,x+1) and $T_{xt}$ is the total number of years lived by this synthetic cohort during and after this age interval. In Section 3, we calculate life expectancies at birth and at age 65, i.e., $e_{0t}$ and $e_{65t}$, respectively

The modal age of death is age-associated with the maximum frequency of death from the synthetic cohort experiencing mortality rates for year *t*. Its expression according to [30] is,

$$M\left(t\right)=\{x|\max\left[d_{xt}\right]\;for\;x>5\}.$$

This indicator’s choice is justified because it can reflect changes in the probability of death $q_{xt}$, which are not detected with life expectancy [31].

However, life expectancy and the modal age of death are both measures of the “typical” length of life of the synthetic cohort, and so do not provide any information about whether changes in mortality apply equally to different age groups. In contrast, the Gini coefficient can be used as a measure of inequality in the length of life, as discussed in [32], as well as being the most common statistical index of diversity or inequality for other variables in the social sciences. Other indices such as the Interquartile range (IQR), which also allow the measurement of this unequal contribution, do not have some desirable basic properties for measuring inequality [32].

The Gini index is calculated using the area underneath the Lorenz curve for the distribution of time of death, which is the curve obtained when we plot the proportion of our synthetic cohort who have died before age *x*, $f_{xt}$, given by

$$f_{xt}=\frac{l_{0t}-l_{xt}}{l_{0t}}=1-\frac{l_{xt}}{l_{0t}},$$

on the *x*-axis, and the cumulative proportion of the years lived by this (deceased) population compared with the total for the synthetic cohort, $g_{xt}$, given by

$$g_{xt}=\frac{T_{0t}-T_{xt}-xl_{xt}}{T_{0t}},$$

on the *y*-axis. By definition, $0\leq f_{xt}\leq 1$ and $0\leq g_{xt}\leq 1$, with $f_{0t}=g_{0t}=0$ and $f_{\omega t}=g_{\omega t}=1$, where ω is the oldest age in the life table. Furthermore, the Lorentz curve is always below the diagonal $f_{xt}=g_{xt}$ with equality only in the case where the entire cohort dies at one specific age. For discrete life tables, one of the most widely used approaches for estimating the Gini index is,

$$I_{G_{t}}=\frac{\sum_{x=0}^{\left(\omega -1\right)}\left(f_{xt}-g_{xt}\right)}{\sum_{x=0}^{\left(\omega -1\right)}f_{xt}},$$

where ω is the last age observed.

Hence, the Gini index measures the dispersion of deaths in the synthetic cohort across the age range. It varies from 0, meaning that all individuals die at the same age, to 1, where almost the entire cohort dies at birth except one individual who dies at age ω. [7] found it to be an excellent indicator to discriminate European Union countries.

### 2.3. Block-Bootstrap Prediction Intervals

Forecasts of the mortality indicators in the future are highly uncertain. Therefore, we illustrate this uncertainty by calculating confidence intervals for the forecast indicators. To consider parameter error, several different bootstrapping procedures have been proposed in [33,34].

In the case where the residuals from the fitting model have dependence across two-dimensions (i.e., age, and time), the ordinary bootstrap in [34] is not valid. Therefore, we use a residual-based block-bootstrap of the fitted residuals, as proposed in [8], because this technique partially retains the underlying dependence structure observed in the residuals and hence generates more realistic resamples [35].

The models in this paper are fitted using the quasi-binomial model. Therefore, the deviance residuals from fitting the model to data are given by expression (Equation (2))

(2) $$r_{xt}=sign\left(d_{xt}-{\hat{d}}_{xt}\right)\sqrt{2\left[d_{xt}\log\left(\frac{d_{xt}}{{\hat{d}}_{xt}}\right)+\left(E_{xt}-d_{xt}\right)\log\left(\frac{E_{xt}-d_{xt}}{E_{xt}-{\hat{d}}_{xt}}\right)\right]},$$

where $d_{xt}$ is the observed and ${\hat{d}}_{xt}$ is the fitted number of deaths for age *x* and year *t*.

The block-bootstrap procedure starts with the rectangular array of deviance residuals, $r_{xt}$, with age *x* in rows, and calendar time *t* in columns. To obtain a new artificial set of residuals, ${\hat{r}}_{xt}^{\left(n\right)}$, we partition an empty rectangular array, with the same dimensions as the original matrix of residuals, into smaller, non-overlapping rectangular blocks. Each block is then filled by a randomly selected block of residuals from the original array. This randomly selected block consists of all residuals in the rectangle to the southeast from a randomly selected element from the original matrix.

To select the appropriate block size, in the absence of firm theoretical guidance, [8] plot correlograms and contour maps of the original residuals and compare these with the resampled residuals. If these plots match reasonably well, this suggests that there is a similar underlying dependence structure in the resampled residuals and in the original set. In this paper, our initial guesses are based on the dependence structure observed for the contour maps of variograms or covariance function of the raw residuals. The observation of these maps allows us to identify the distance in years and age from which we can admit the independence between the residuals, and these values define the dimensions of the block. Further details can be found in [10]. These resampled residuals, ${\hat{r}}_{xt}^{\left(n\right)}$, are then combined with the fitted death counts, ${\hat{d}}_{xt}$, from the original model, by solving

(3) $${\left({\hat{r}}_{xt}^{\left(n\right)}\right)}^{2}=2\left[d_{xt}\log\left(\frac{d_{xt}}{{\hat{d}}_{xt}}\right)+\left(E_{xt}-d_{xt}\right)\log\left(\frac{E_{xt}-d_{xt}}{E_{xt}-{\hat{d}}_{xt}}\right)\right],$$

to give resampled “observations” of the death counts, the modified $d_{xt}^{\left(n\right)}$. These resampled death counts are then fitted using one of the three mortality models being considered, providing new estimates of the model parameters.

However, it should not be essential that the model used to fit these resampled death counts is the same as the original model used to generate the residuals and fitted mortality rates. Therefore, there are nine possible combinations of models given by the three models used to generate the residuals and fitted mortality rates used by the block-bootstrap technique. The process is repeated to give *N* bootstrap samples of death counts, which, in turn, provide *N* resampled sets of model parameters. For each of these, the $k_{t}^{\left(i\right)}$s and $\gamma_{t-x}$s are projected on the basis of an ARIMA model selected using the Box-Jenkins procedure. Hence, we obtain predictions for the mortality rates and the corresponding mortality indicators for desired future years allowing fully for parameter error. The 95% confidence intervals, $IC_{95}$, for the different mortality indicators are obtained by selecting the 2.5% and 97.5% percentiles of the projected indicators for the desired future years, for example, $IC_{95}^{e_{0,2020}}=\left[p_{0.025}^{e_{0,2020}},p_{0.975}^{e_{0,2020}}\right]$ represents the 95% confidence interval for period life expectancy at birth in 2020, based on the 2.5% and 97.5% percentiles of the projected distribution for this indicator.

### 2.4. ANOVA for Functional Data Analysis

The experimental design we have just described is a two fixed factor design: the model used for fitting, and the sample obtained from the residual used for resampling. Its structure is shown in Table 1.

It is a balanced design with the same number of repetitions in each cell, *N*, equal to the number of block-bootstrap samples. Each of these repetitions is 20 values, the projected mortality indicator corresponding to the years 2013 to 2032. The factor *model* has three categories, the Lee-Carter with one time-dependent term, *LC1*, the Lee-Carter with two time-dependent terms, *LC2*, and the Lee-Carter with one time-dependent term and one cohort term, *H1*. In turn, the factor *sample* also has three categories reflecting the origin of the fitted and residuals used in the bootstrap process, *RLC1*, *RLC2* and *RH1* according to whether they were obtained from the fitting of the original data with the *LC1*, *LC2* or *H1* model.

As mentioned at the beginning, we aim to check differences among the projections obtained with each combination of model and block-bootstrap sample. A classic ANOVA method should not be applied in this context. We have to deal with functional data, and the use of functional data analysis methods is a natural way to proceed. Several authors have dealt with the development of ANOVA techniques for these kinds of data, but not all of these methods can be used in a design with two fixed factors. Some of them address a single factor [36,37] and others address mixed-effects [38,39]. Therefore, we will resort to the method proposed by [40], which is an effective, flexible, and easy to compute technique able to deal with complicated ANOVA designs requiring no normality assumptions and as few additional hypotheses as possible. The method uses random projections to transform functional data into univariate data, solves the ANOVA problem in this simple situation, and obtains conclusions for the functional data by collecting the information from several projections. Following [40], we can decompose any functional data $X_{i}^{mod,sam}\left(t\right)$ as,

(4) $$X_{i}^{mod,sam}\left(t\right)=m\left(t\right)+f^{mod}\left(t\right)+g^{sam}\left(t\right)+h^{mod,sam}\left(t\right)+\epsilon_{i}^{mod,sam}\left(t\right),$$

where *m* is non-random and describes the overall shape of the projections, i=1,…,N, and the functions $f^{mod}$, $g^{sam}$ and $h^{mod,sam}$ account for the main effect and interaction of model and sample. Finally, $\epsilon_{i}^{mod,res}$ are independent and identically distributed random trajectories centered on the mean.

## 3. Results

The data used in this analysis come from the life tables published by the Spanish National Institute of Statistics (INE) and are available on its web page www.ine.es (accessed on 15 May 2020). In particular, we have worked with the crude estimates of probabilities of death, $q_{xt}$, obtained using the methodology proposed by [41], based on [42]. This more recent Spanish dataset is computed using micro-mortality data, based on individual dates of death, and with no smoothing procedure applied at the oldest ages. Therefore, this dataset is more accurate than those provided by the Human Mortality Database [41]. Also, this paper describes the steps that are taken, and R-packages are quoted for the sake of replicability and reproducibility.

### 3.1. Model Fitting

The models described in Section 2.1 (i.e., the *LC1*, *LC2* and *H1* models) have been used to fit mortality data for Spain for the period 1991–2012 and ages 0 to 99. As discussed in Section 2.1, the estimation of the parameters for these models is carried out by means of maximum likelihood methods assuming the quasi-binomial distribution for the death counts, using the gnm R-package [21].

However, the models proposed in Section 2.1 are over-parameterised, and require additional identifiability constraints in order to obtain unique estimates of the parameters. The model *LC1* is usually fitted with the location constraint $\sum_{t}k_{t}^{\left(1\right)}=0$ and the scale constraint $\sum b_{x}^{\left(1\right)}=1$ [3,27]. However, in this paper, we use the alternative constraints $k_{t_{0}}^{\left(1\right)}=0$ and $\sum b_{0}^{\left(1\right)}=1$, because these are simple to be specified using the gnm function with the additional constrain and constrainTo inputs. In addition, we have used the residSVD function to provide initial estimates of the parameters, to speed up and ensure convergence of the fitting algorithm.

In a similar manner, the *LC2* model can be fitted with the gnm library using the instances function. For consistency with the *LC1* model, we apply the restrictions $k_{t_{0}}^{\left(1\right)}=0$, $\sum b_{0}^{\left(1\right)}=1$, $k_{t_{0}}^{\left(2\right)}=0$ and $\sum b_{0}^{\left(2\right)}=1$.

For the *H1* model, the issue of identifiability constraints becomes more difficult due to the relationship between the factors, cohort=period−age, as noted by [25]. To overcome this, [24] propose carrying the estimation out in two stages. First, $a_{x}$ is fixed, in a similar manner as was originally done in [3], i.e.,

(5) $${\hat{a}}_{x}=\frac{\sum_{t}\ln\left(\frac{q_{xt}}{1-q_{xt}}\right)}{T}.$$

Then, the remaining parameters can be estimated with the fixed values of $a_{x}$ by using the offset term in the gnm function. In this paper, for the *H1* model, we further impose the constraints $k_{t_{0}}^{\left(1\right)}=0$, $\sum b_{0}^{\left(1\right)}=1$ to identify the model and an additional constraint, $b_{x}^{\left(1\right)}>0\;\forall x$, to ensure that mortality rates are positively correlated across the age range. Furthermore, we have set the weight of the first and last five cohorts to zero, as done in [24], to avoid estimating parameters for which we have very little data.

**Figure 2:**
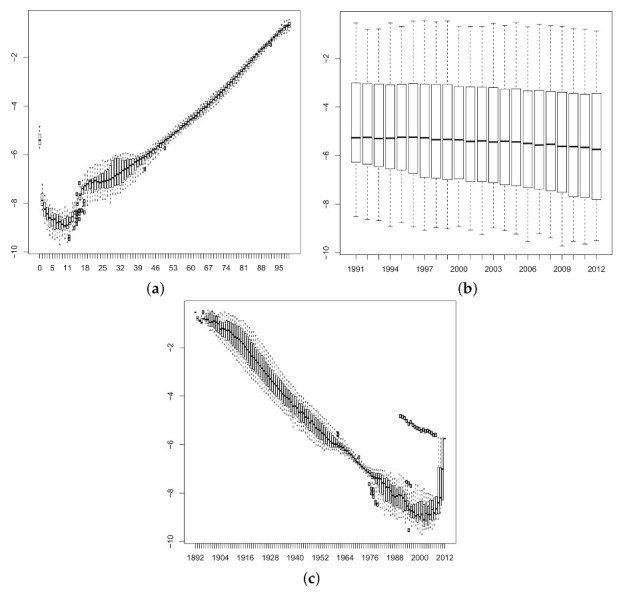
Behaviour of the logit of the crude mortality rates. (**a**) for age, (**b**) time and (**c**) cohort.

For the sake of brevity, we have only fitted data for males and have not reproduced our results for females. Figure 2a–c show the behaviour of the logit of the crude mortality rates according to age *x*, period *t* and cohort t−x, respectively, by grouping the crude mortality rates by the factors age, period and cohort and plotting box-and-whisker diagrams them. We note that Figure 2c shows outliers for cohorts born in 1990 until 2009, which correspond to the high rates of mortality at birth. The goodness of fit of these models to the data is evaluated using the total deviance, which is shown for all three models with the corresponding number of parameters in Table 2.

**Figure 3:**
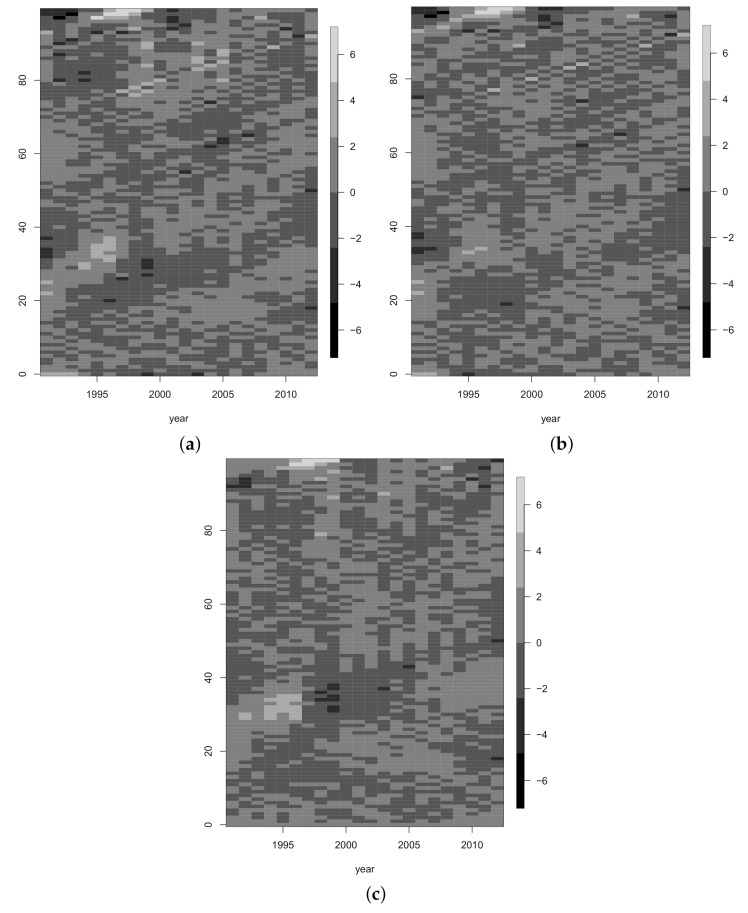
Residuals for age-period for each model for males. (**a**) *LC1*, (**b**) *LC2* and (**c**) *H1*.

Additionally, [25] suggest carrying out diagnostic checks on the fitted model by plotting residuals. Figure 3a–c show the underlying dependence structure in the deviance residuals given by expression (Equation (2)) for *LC1*, *LC2* and *H1*, respectively. As can be seen in Figure 3c, the inclusion of the cohort effect for model *H1* has partially eliminated the diagonal effect appearing in the other two models. However, the residuals for any of the models still show significant two-dimensional dependence.

### 3.2. Prediction Intervals for Mortality Indicators

As discussed in Section 2.3, the standard bootstrap of [34] is not valid in the case where the residuals show two-dimensional dependence, since it is based on simple random sampling. Instead, use the block-bootstrap technique suggested by Liu [8]. To determine the optimum block sizes, we analyse the dependence structure observed for the residuals in Figure 3a–c using a variogram [10]. These plots show large patches of positive and negative values (some of which are of very large magnitude), which is suggestive of a high degree of two-dimensional dependence and so requires the use of large block sizes.

Therefore, we use block sizes of 3×9, 3×9 and 3×14 for *LC1*, *LC2* and *H1*, respectively. As described in Section 2.3, we then use these resampled death counts from the bootstrapping procedure to refit the models and then forecast confidence intervals for the mortality indicators for the period 2013–2032. In general, these confidence intervals show that life expectancy at birth, residual life expectancy at age 65, and the modal age of death continue to increase, and the forecast Gini index decreases.

Table 3 shows the INE estimations and the corresponding confidence intervals for *LC1* for predicted life expectancy at birth and at 65, noting that the *LC1* model furnishes the highest values and the values closest to the INE ones.

**Figure 4:**
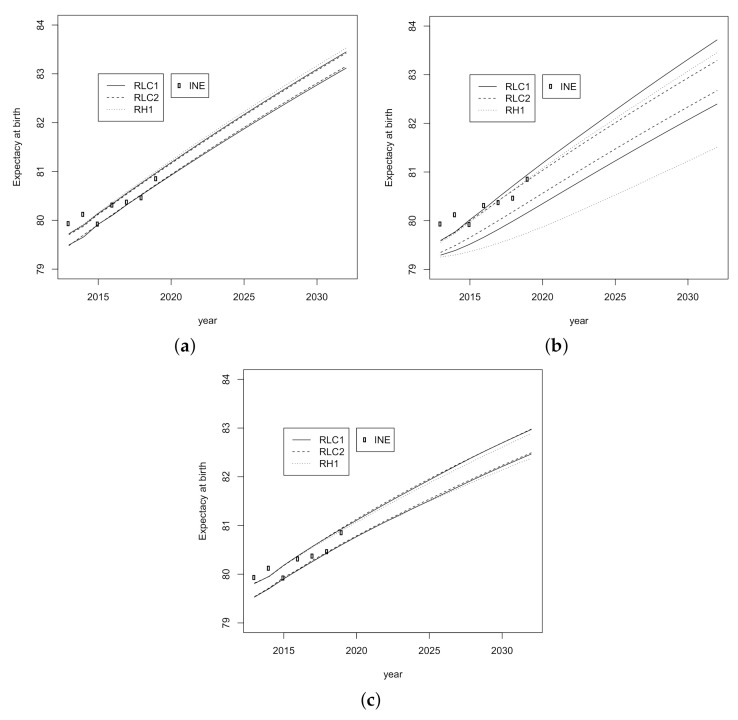
Confidence intervals for forecasted Spanish life expectancy for the period 2013–2032. (**a**) *LC1*, (**b**) *LC2* and (**c**) *H1*.

Confidence intervals for forecasted Spanish life expectancy at birth for the period 2013–2032 are shown in Figure 4 where the interval corresponding to *LC2* model is the widest. In addition, life expectancies for the period 2013–2019 published by INE are outside of all intervals for the first two years (2013 and 2014).

**Figure 5:**
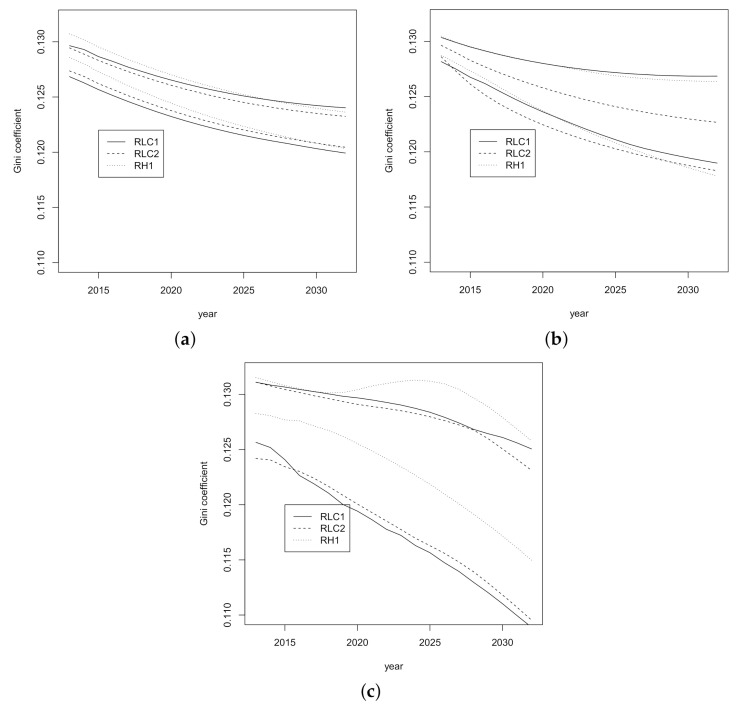
Confidence intervals for forecasted Gini index for the period 2013–2032. (**a**) *LC1*, (**b**) *LC2* and (**c**) *H1*.

Confidence intervals for forecasted Gini index for the period 2013–2032 are shown in Figure 5 where the interval corresponding to the *H1* model is the widest. Also, the *H1* model shows the Gini index with greatest decrease.

### 3.3. ANOVA for Functional Data Analysis

We use the functional ANOVA procedure to test differences in the behavior of the mortality indicators for the nine different combinations of the model and the block-bootstrap sample. Thus, we expect a model effect, but no sample or interaction effects since the samples represent the same population regardless of the model that generated them.

Using Equation (4), we test the following null hypotheses

$$\begin{array}{ccc} H_{0}^{mod} & : & f^{LC1}=f^{LC2}=f^{H1}=0\\ H_{0}^{sam} & : & g^{RLC1}=g^{RLC2}=g^{RH1}=0\\ H_{0}^{mod,sam} & : & h^{model,sample}=0,\;\;model=\left\{LC1,LC2,H1\right\},\\ & & residual=\{RLC1,RLC2,RH1\}. \end{array}$$

for each indicator, the alternative hypothesis being that some of the equalities are not true. As discussed in Section 2.4, the technique we use to do this was introduced by [40]. These authors have also developed an R-package [43], which obtains p-values by three different approaches: the Bonferroni method, the false discovery rate method and the bootstrap method. As [43] point out, the Bonferroni correction is conservative. In contrast, the bootstrap method is time consuming, and requires proofs to show that it is appropriate in the specific contexts in which it is applied to. Therefore, the we use the false discovery rate (FDR) method, proposed by [44].

Table 4 summarizes the result of the functional ANOVA obtained with the False Discovery Rate method, where *NR* stands for the non-rejection of the null hypothesis and *R* for rejection.

**Figure 6:**
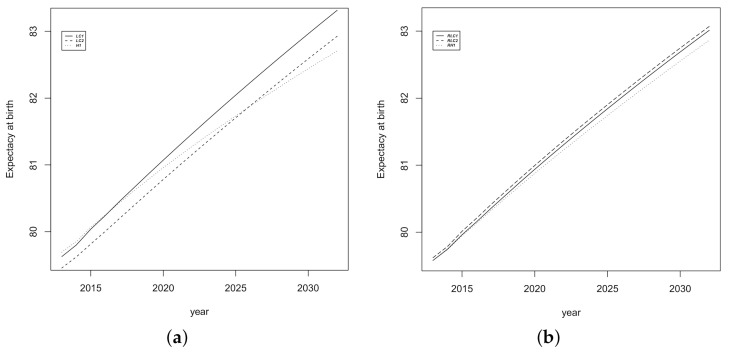
Average forecasting estimations for life expectancy. (**a**) Model (**b**) Sample.

**Figure 7:**
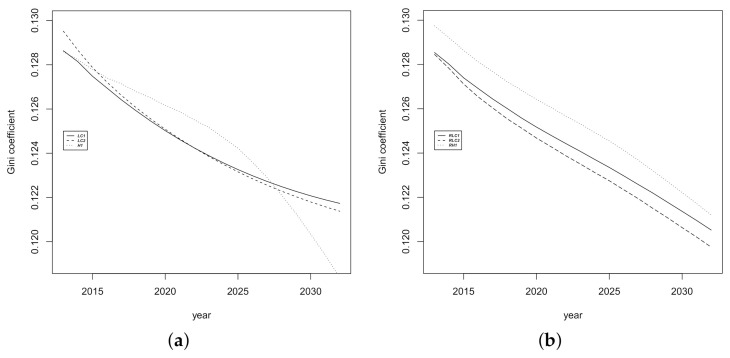
Average forecasting functions for the Gini index. (**a**) Model (**b**) Sample.

We have also performed multiple comparisons to establish whether there are homogeneous groups among the categories of the main factors tested above. The results of these are shown in Table 5. Figure 6 and Figure 7 help us to understand the results in Table 5. From Figure 6a, we note that *LC1* model provides greater projections of the life expectancy than the other two models, and Figure 6b displays small but significant differences between the bootstrap samples. As regards the Gini index, the *LC1* and *LC2* models show a similar trend over time, but there is a more pronounced decrease corresponding to the *H1* model (Figure 7a). For the sample factor, the lowest values of the Gini index corresponds to the *RLC1* sample of residuals whose projections are significantly different from the other two (Figure 7b). The equivalent graphs for the other two indicators (life expectancy at age 65 and modal age at death) are not reproduced here for the sake of brevity. The interaction between the model and the residuals deserves particular comment.

## 4. Conclusions

In terms of the key indices of interest for mortality forecasting, specially in an actuarial context, refs. [10,13,14,24,45,46] considered death rates, life expectancies and discounted annuity values. This paper evaluates if differences between three different extensions of the Lee-Carter model are reflected in the forecasts of different mortality indicators. The three mortality indicators used are the life expectancy and modal age of death (which are measures of typical life span in a population) and the Gini index (which is a measure of the dispersion of life span). As far as we are aware, there are no previous studies to date on the impact of model risk on forecasting of the Gini index. To illustrate the uncertainty in our forecasts, we calculate confidence intervals for the forecast indicators using the block-bootstrap technique proposed in [8] for the fitted residuals.

To evaluate whether the differences in the forecast mortality indicators between the different mortality models are statistically significant, we have used functional ANOVA to test the two-factor design resulting from crossing models and block-bootstrap samples, as discussed in Section 2.3 and Section 2.4. In Table 4, we show a statistically significant model, sample, and interaction effects between these two factors for all of the mortality indicators under consideration. Although it was not our main objective, it is essential to point out that the sample effect should not be present since they must be realizations of the same population, and therefore the procedure should lead to samples that do not influence the results. Most authors test their models with the samples derived from their fit, leading to biased conclusions from their results.

We find that our predictions for $e_{0t}$ and $e_{65t}$ with Spanish mortality data are higher than those obtained in previous studies [6,10,23]. Also, there are significant differences in the forecasts of all of the indicators between the different models which we investigate in this study. These differences in forecasts between models is a key component of the “longevity risk”, as identified by the IMF in [47]. Larger predictions may be more realistic than those obtained previously and may represent a better response to the financial challenge that “longevity risk” implies, as noted by the IMF in a recent report [47]. Therefore, in terms of longevity risk, when different models disagree, the preferred model could be the one with the greatest predicted life expectancy, which in our case is *LC1*. Although the addition of further terms improves the adjustment of probabilities and translates into an effect on any derived mortality indicators, the models’ predictions must be checked in terms of their probabilities and the mortality indicators obtained.

In this study, we use the functional ANOVA to provide an objective criterion for measuring the impact of different techniques for forecasting mortality indicators. This technique is complementary to measures such as goodness-of-fit statistics, as used in [13,14,24]. Although the conclusions that we reach are based on the dataset for Spanish males, the use of the block-bootstrap and the statistical tools proposed provide a framework for investigating a wide range of mortality modeling hypotheses. However, we leave it to future work to look at other datasets to examine whether our conclusions are consistent for other populations and to draw more general conclusions about the impact of model risk on the forecasting of mortality indicators.

## Figures and Tables

**Table 1:** Experimental design for comparison of functional indicators.

		Model		
		*LC1*	*LC2*	*H1*
	*LC1*	N	N	N
Sample	*RLC2*	N	N	N
	*RH1*	N	N	N

**Table 2:** Goodness-of-fit measures for the different models.

	*LC1*	*LC2*	*H1*
Deviance	2911.45	2136.05	2192.48
Number of parameters	100 + 22 + 100	100+22×2+100×2	100 + 22 + 100 + 121

**Table 3:** Confidence intervals for forecasted Spanish life expectancy for the period 2013–2032 using *LC1* only for men.

	Life Expectancy at Birth							Life Expectancy at 65						
		*RLC1*		*RLC2*		*RH1*			*RLC1*		*RLC2*		*RH1*	
Year	INE	p${}_{0.025}$	p${}_{0.975}$	p${}_{0.025}$	p${}_{0.975}$	p${}_{0.025}$	p${}_{0.975}$	**INE**	p${}_{0.025}$	p${}_{0.975}$	p${}_{0.025}$	p${}_{0.975}$	p${}_{0.025}$	p${}_{0.975}$
2013	79.93	79.46	79.73	79.49	79.68	79.49	79.71	18.92	18.59	18.76	18.62	18.72	18.62	18.74
2014	80.12	79.64	79.90	79.70	79.84	79.65	79.89	19.06	18.71	18.86	18.73	18.82	18.72	18.86
2015	79.92	79.87	80.14	79.92	80.08	79.90	80.12	18.79	18.84	19.01	18.87	18.97	18.87	19.00
2016	80.31	80.06	80.35	80.13	80.29	80.10	80.33	19.14	18.97	19.14	19.00	19.10	18.99	19.14
2017	80.37	80.27	80.57	80.34	80.50	80.31	80.55	19.12	19.10	19.27	19.13	19.24	19.12	19.27
2018	80.46	80.47	80.77	80.54	80.71	80.51	80.76	19.22	19.23	19.41	19.26	19.37	19.25	19.41
2019	80.85	80.66	80.98	80.74	80.92	80.72	80.97	19.52	19.35	19.55	19.39	19.50	19.38	19.54
2020		80.86	81.19	80.94	81.12	80.92	81.18		19.48	19.68	19.52	19.63	19.51	19.67
2021		81.05	81.39	81.14	81.32	81.12	81.38		19.61	19.82	19.64	19.77	19.64	19.81
2022		81.24	81.59	81.33	81.52	81.31	81.58		19.73	19.95	19.77	19.90	19.76	19.94
2023		81.43	81.78	81.53	81.72	81.50	81.77		19.86	20.09	19.90	20.03	19.89	20.07
2024		81.62	81.98	81.72	81.91	81.69	81.97		19.98	20.22	20.02	20.16	20.02	20.20
2025		81.80	82.17	81.91	82.10	81.88	82.16		20.11	20.35	20.15	20.29	20.14	20.33
2026		81.99	82.37	82.10	82.29	82.07	82.36		20.23	20.49	20.27	20.42	20.27	20.46
2027		82.17	82.56	82.28	82.47	82.25	82.54		20.35	20.62	20.40	20.55	20.39	20.59
2028		82.35	82.75	82.46	82.66	82.43	82.73		20.48	20.75	20.52	20.67	20.51	20.72
2029		82.52	82.93	82.64	82.84	82.61	82.92		20.60	20.88	20.64	20.80	20.64	20.85
2030		82.70	83.11	82.81	83.02	82.79	83.10		20.72	21.00	20.77	20.93	20.76	20.98
2031		82.87	83.29	82.99	83.20	82.96	83.28		20.84	21.13	20.89	21.05	20.88	21.11
2032		83.04	83.47	83.16	83.37	83.14	83.46		20.96	21.26	21.01	21.18	21.00	21.23

**Table 4:** Rejection (R) or non-rejection (NR) of null hypothesis with functional ANOVA with FDR.

	Model	Residual	Model × Residual
Indicator	$H_{0}^{\mathrm{mod}}$	$H_{0}^{\mathrm{sam}}$	$H_{0}^{\mathrm{mod},\mathrm{sam}}$
$e_{0t}$	R	R	R
$e_{65t}$	R	R	R
Gini index	R	R	R
Modal age	R	R	R

**Table 5:** Multiple comparison for Models and Samples.

	**Model**		
	$f^{\mathrm{LC}1}=f^{\mathrm{LC}2}$	$f^{\mathrm{LC}1}=f^{H1}$	$f^{\mathrm{LC}2}=f^{H1}$
$e_{0t}$	R	R	R
$e_{65t}$	R	R	R
Gini index	R	R	R
Modal age	R	R	R
	**Sample**		
	$g^{\mathrm{RLC}1}=g^{\mathrm{RLC}2}$	$g^{\mathrm{RLC}1}=g^{\mathrm{RH}1}$	$g^{\mathrm{RLC}2}=g^{\mathrm{RH}1}$
$e_{0t}$	R	R	R
$e_{65t}$	R	R	R
Gini index	R	R	R
Modal age	R	R	R

## Data Availability

The data used in this analysis come from the life tables published by the Spanish National Institute of Statistics (INE) and are available on its web page www.ine.es (accessed on 15 May 2020).
